# Effects of Sanghuang Extract on *Candida albicans* Colonization and Intestinal Inflammation in a DSS-Induced Colitis Mouse Model

**DOI:** 10.3390/pathogens15101071

**Published:** 2026-10-09

**Authors:** Zesen Fang, Zhijia Dong, Silin Liu, Haonan Dong, Yang Yang, Jianghan Luo, Lijun Yan, Yuan Gao

**Affiliations:** School of Pharmacy, Engineering Research Center of Natural Antineoplastic Drugs, Ministry of Education, Harbin University of Commerce, Harbin 150076, China; fangzeseny024@s.hrbcu.edu.cn (Z.F.); tyharuka0930@163.com (Z.D.); liusilin@s.hrbcu.edu.cn (S.L.); donghaonany025@s.hrbcu.edu.cn (H.D.); yangyang@hrbcu.edu.cn (Y.Y.); 102690@hrbcu.edu.cn (J.L.); ylj@hrbcu.edu.cn (L.Y.)

**Keywords:** Sanghuang extract, *Candida albicans*, ulcerative colitis, intestinal bacterial community, PI3K/AKT/mTOR pathway

## Abstract

*Sanghuangporus sanghuang* (Sanghuang) extract, obtained from the medicinal and edible fungus Sanghuang, was examined for its inhibitory effects against *Candida albicans* and its potential role in mitigating intestinal inflammation related to ulcerative colitis (UC). Its anti-*Candida albicans* activity was assessed by measuring the minimum inhibitory concentration (MIC), evaluating its ability to suppress biofilm formation, and observing fungal morphological changes using scanning electron microscopy (SEM). Using mice with dextran sulfate sodium (DSS)-induced UC and intestinal *Candida albicans* (*C. albicans*) colonization, fungal burden, intestinal inflammation, mucosal injury, intestinal bacterial community composition, and related signaling pathways were assessed. Sanghuang extract inhibited *C. albicans* growth and suppressed biofilm formation. SEM further revealed reduced hyphal development and disruption of cell surface morphology following treatment. In vivo, Sanghuang administration ameliorated UC-related phenotypes, with descriptive decreasing trends in fecal *C. albicans* burden, less severe colonic injury, and lower colonic levels of IL-6, IL-1β, and TNF-α, accompanied by changes in intestinal bacterial community composition. Network pharmacology analysis suggested that pathways associated with phosphoinositide 3-kinase (PI3K)/protein kinase B (AKT) may participate in this process. Molecular docking yielded predicted interactions of caffeic acid with PTGS2, EGFR, PIK3CA, AKT1, and mTOR. qRT-PCR further showed that Sanghuang extract reduced the colonic mRNA expression of *Pik3ca*, *Akt1*, and *Mtor*. These findings provide experimental evidence that Sanghuang extract exerts antifungal activity in vitro and is associated with changes in fecal *C. albicans* burden and colitis-associated inflammatory responses in this mouse model.

## 1. Introduction

Ulcerative colitis (UC) represents a recurrent and chronic nonspecific inflammatory condition characterized by mucosal involvement of the colon and rectum [1]. Its clinical management remains challenging because of its persistent course, high recurrence rate, and limited likelihood of complete and durable remission. Although the mechanisms underlying UC have not been fully defined, current evidence suggests that its pathogenesis is driven by multiple interacting factors, including aberrant immune responses, disruption of intestinal epithelial barrier integrity, environmental influences, and microbial dysbiosis [2]. Notably, growing attention has been paid to alterations in the gut microbiome, in which shifts in both bacterial and fungal populations may contribute to disease onset, inflammatory progression, and mucosal injury in UC [3].

As an opportunistic fungal pathogen, *C. albicans* can asymptomatically colonize the gastrointestinal tract under physiological conditions [4]. Under conditions of disrupted intestinal homeostasis, however, this commensal-to-pathogenic transition may be promoted, leading to fungal overgrowth, biofilm formation, filamentous growth, and increased inflammatory injury to the intestinal mucosa. In parallel, dysbiosis of the intestinal microbiota and compromised mucosal barrier integrity may further support persistent *C. albicans* colonization, thereby amplifying local inflammatory responses and contributing to the progression of colitis [5]. These observations suggest that suppressing *C. albicans* overgrowth while restoring gut microecological equilibrium may be a feasible therapeutic approach for UC associated with *C. albicans* infection [6].

Sanghuang, a medicinal fungus, has been reported to possess diverse bioactivities, such as inflammation-relieving, antioxidative, immune-regulatory, and antimicrobial properties [7]. Previous studies have shown that Sanghuang contains various bioactive constituents, especially polyphenolic compounds, which may contribute to its biological activities [8]. In the present study, active components of Sanghuang were screened by database retrieval and literature mining, and six constituents were qualitatively detected in the extract by UPLC. Network pharmacology analysis suggested that Sanghuang may regulate multiple targets and pathways related to inflammation and immune responses, including the PI3K/Akt, MAPK, and PD-L1 signaling pathways. However, the effects of Sanghuang extract on *C. albicans*-associated UC, as well as its possible associations with intestinal bacterial community changes and host signaling responses, remain unclear.

Therefore, in this study, we investigated the inhibitory activity of Sanghuang extract against *C. albicans* in vitro and further evaluated its effects in a mouse model of DSS-induced colitis with intestinal *C. albicans* colonization. In addition, gut bacterial community analysis, network pharmacology, molecular docking, and qRT-PCR were integrated to explore potential associations with host inflammatory and pathway-related responses underlying the protective effects of Sanghuang extract. Previous work has reported antifungal effects of Sanghuang extracts against *C. albicans*, and our group has previously investigated cinnamon essential oil in a related DSS/*C. albicans* model. The present study differs by integrating chemical characterization of Sanghuang extract with in vitro antifungal phenotypes, intestinal *C. albicans* colonization in DSS-induced colitis, bacterial community profiling, network pharmacology, molecular docking, and pathway-related gene-expression analysis.

## 2. Materials and Methods

### 2.1. Materials

Professor Changbin Chen at Shanghai Institute of Immunity and Infection, Chinese Academy of Sciences, generously provided the *C. albicans* strain SN250 (strain No. 250). Fruiting bodies of *Sanghuangporus sanghuang* used in this study were derived from strain GY-S01 obtained from Shandong Guyuan Co., Ltd. (Dezhou, China). The strain is deposited in the China General Microbiological Culture Collection Center (CGMCC) under accession No. 13579. Liaoning Changsheng Biotechnology Co., Ltd. (Benxi, China) supplied the specific pathogen-free (SPF) male BALB/c mice weighing 20 ± 2 g, which were certified as qualified by the Liaoning Provincial Laboratory Animal Quality Testing Center (license no. SCXK (Liao) 2020-0001). The Experimental Animal Ethics Committee of Harbin University of Commerce approved all animal procedures (approval no. HSDYXY-2024092; approval date: 2 December 2024).

### 2.2. Preparation of Sanghuang Extract

The fruiting bodies of Sanghuang were pulverized and passed through a 60-mesh sieve. The resulting powder was extracted by heat reflux with 70% ethanol at a solid-to-solvent ratio of 1:25 (g/mL) at 80 °C for 60 min. The extraction procedure was repeated four times. The resulting extracts were concentrated, the solvent was removed, and the residue was thoroughly dried to obtain the Sanghuang ethanol extract used in the subsequent experiments. The dried extract was stored at −20 °C in airtight containers protected from light until use.

The reproducibility of the optimized extraction procedure was evaluated using three parallel extractions. Total polyphenol content was determined using gallic acid as the reference standard. The calibration curve was Y = 124.0X + 0.02173 (R^2^ = 0.9992). The total polyphenol content obtained under the optimized extraction conditions was 11.009 ± 0.22 mg gallic acid equivalents (GAE)/g extract (*n* = 3), with an RSD of 1.96%.

### 2.3. UPLC Analysis of Sanghuang Extract and Standard Compounds

Caffeic acid, protocatechuic aldehyde, myricitrin, p-coumaric acid, and luteolin reference standards (0.1 mg each), as well as kaempferol-3-*O*-rutinoside (0.2 mg), were accurately weighed and separately dissolved in 1 mL of 80% aqueous methanol. The solutions were ultrasonicated until completely dissolved and filtered through 0.22 μm membranes to prepare individual stock solutions at concentrations of 0.1 mg/mL for caffeic acid, protocatechuic aldehyde, myricitrin, p-coumaric acid, and luteolin, and 0.2 mg/mL for kaempferol-3-*O*-rutinoside. Subsequently, 100 μL of each stock solution was accurately transferred and combined, and the mixture was diluted to 1 mL with 80% aqueous methanol to prepare the mixed standard solution. Sanghuang extract (1 mg) was dissolved in 80% methanol–water to 1 mg/mL and filtered to obtain the test solution. Chromatographic separation was performed on a Waters Acquity UPLC BEH C18 column at 325 nm using a gradient of acetonitrile and 0.1% aqueous formic acid [9]. The standard and sample solutions were then analyzed to compare the chromatographic profiles [10]. The UPLC analysis was used for qualitative identification of the detected constituents by comparison with reference standards. Method precision, stability, and repeatability were evaluated, with RSD values generally below 3%. However, compound-specific calibration curves and recovery experiments were not performed; therefore, the six detected constituents were not reported as quantitatively validated concentrations.

### 2.4. Antifungal Evaluation of Sanghuang Extract Against C. albicans In Vitro

Frozen stocks of *C. albicans* SN250 were thawed and streaked onto yeast extract peptone dextrose (YPD) agar containing 20 g/L glucose, 20 g/L peptone, 10 g/L yeast extract, and 20 g/L agar, followed by incubation at 30 °C for 1–2 days. A single colony was transferred into YPD broth containing 20 g/L glucose, 20 g/L peptone, and 10 g/L yeast extract and cultured at 30 °C for 24 h with shaking at 120 rpm. Cell density was determined using a hemocytometer, and the fungal suspension was adjusted to 2 × 10^6^ CFU/mL before use.

MIC_80_ was determined using a broth microdilution method adapted from the CLSI M27-A guideline, with modifications to the culture medium, inoculum density, incubation temperature, and incubation time. Briefly, 100 μL of each Sanghuang extract or clotrimazole solution was added to a 96-well plate, followed by 100 μL of a *C. albicans* SN250 suspension at 2 × 10^6^ CFU/mL, resulting in a final fungal inoculum of 1 × 10^6^ CFU/mL per well. Clotrimazole (Aladdin, Shanghai, China) was first dissolved in dimethyl sulfoxide (DMSO) to prepare a stock solution and was subsequently diluted with sterile saline to the required working concentrations before use. Sterile saline was used in the untreated growth-control wells. Fungus-free wells containing YPD broth were used as blank controls, and corresponding drug-containing wells without fungal cells were included to correct for background absorbance from the test solutions. Plates were incubated at 30 °C for 48 h, and absorbance was measured at 630 nm. MIC_80_ was defined as the lowest tested concentration producing at least 80% inhibition of fungal growth relative to the untreated growth control. The MIC assay was independently repeated three times (*n* = 3 independent experiments).

Time–growth curves were generated by incubating *C. albicans* with Sanghuang extract at 2 × MIC, 1 × MIC, or 0.5 × MIC, or with clotrimazole at 1 × MIC at 37 °C. OD_630_ was measured at regular intervals between 0 and 48 h to monitor growth kinetics [11].

To evaluate the inhibitory effect of Sanghuang extract on *C. albicans* biofilm formation, different concentrations of Sanghuang extract and clotrimazole were prepared and added to 96-well plates (100 μL/well). Subsequently, 100 μL of a *C. albicans* SN250 suspension prepared in RPMI 1640 medium (Servicebio, Wuhan, China) at 2 × 10^6^ CFU/mL was added to each well, resulting in a final fungal inoculum of 1 × 10^6^ CFU/mL. Thus, the test compounds were present throughout the 24 h biofilm-development period. Plates were incubated at 37 °C for 24 h, after which the wells were washed three times with PBS [12]. XTT/menadione solution (200 μL; final menadione concentration, 10 μM) was then added to each well and incubated at 37 °C for 2 h in the dark. The supernatant absorbance was measured at 490 nm. SMIC_80_ was operationally defined as the lowest tested concentration producing ≥80% inhibition of biofilm metabolic activity relative to the untreated biofilm control. The assay was performed in triplicate (*n* = 3).

Biofilm inhibition rate (%) = [1 − (OD _treatment_/OD _negative control_)] × 100

### 2.5. Effect of Sanghuang Extract on C. albicans Hyphal Morphology

*C. albicans* suspensions were prepared in RPMI 1640 medium and dispensed into 6-well plates at 200 µL per well, followed by pre-incubation at 37 °C for 1 h. Sanghuang extract (1375, 2750 [SMIC], and 5500 µg/mL [2 × SMIC]) or clotrimazole (35 µg/mL) was added, and saline was used in blank controls [13]. Plates were incubated at 37 °C for 12 and 24 h.

For SEM observation, treated cells were pelleted at 3000 rpm for 3 min, followed by overnight fixation with 2.5% glutaraldehyde at 4 °C. The fixed cells were washed in PBS and dehydrated stepwise using an ethanol gradient of 30%, 50%, 70%, 90%, and 100% for 15 min at each concentration. After each dehydration step, the cells were collected by centrifugation at 6000 rpm for 5 min. Finally, the samples were left to dry overnight, sputter-coated with gold, and visualized under a scanning electron microscope (JEOL, Tokyo, Japan) for hyphal ultrastructure examination [14]. The SEM experiment was independently repeated three times.

### 2.6. Establishment of a DSS-Induced Colitis Mouse Model with Intestinal C. albicans Colonization

Specific pathogen-free (SPF) male BALB/c mice (20 ± 2 g) were acclimatized for 7 days under controlled temperature and humidity with a 12 h light/dark cycle, and then randomly allocated into eight groups (*n* = 10 per group): Control, DSS + C.A (UCCA model), DSS, Caffeic Acid, SP-L, SP-M, SP-H, and mesalazine. The DSS + C.A group represents the UCCA model, defined as DSS-induced ulcerative colitis accompanied by intestinal *C. albicans* colonization. For consistency with the graphical group labels, “DSS + C.A” is retained in the figures, whereas “UCCA” is used in the main text when referring to this disease model.

The Control group received distilled water ad libitum throughout the experiment and underwent no gavage or DSS exposure. Mice in the DSS-only group received distilled water ad libitum and sterile saline by oral gavage once daily from day 1 to day 4, followed by 3% DSS (Aladdin, Shanghai, China) solution ad libitum from day 5 to day 11 [15]. The UCCA model group and all intervention groups received distilled water ad libitum and *C. albicans* (1 × 10^8^ CFU/mouse/day) by oral gavage from day 1 to day 4, followed by 3% DSS solution ad libitum from day 5 to day 11 [16]. Mice were euthanized on day 12 for sample collection. Animals were fasted overnight before euthanasia but had free access to drinking fluid; mice undergoing DSS exposure continued to receive the scheduled DSS solution during this period. Body weight was recorded daily from day 1 to day 11. For longitudinal analysis, body weight at each time point was normalized to the day 1 value of the same animal and expressed as a percentage of baseline body weight. The biological replicate number for body weight analysis was *n* = 10 mice per group.

A power analysis was conducted a priori using G*Power version 3.1.9.7. For eight experimental groups, with an effect size of 1.2, α = 0.05, and a power of 0.80, at least eight mice were required per group. After allowing for an estimated 20% attrition, 10 mice were included in each group, resulting in a total of 80 animals. This design provided adequate statistical power while adhering to the reduction principle of the 3R principles. All outcomes were independently evaluated under single-blind conditions, and treatment identities were disclosed only after data acquisition and statistical processing had been completed.

All animals in each group were included for body weight monitoring, colon-length measurement, and fecal *C. albicans* burden assessment (*n* = 10 per group). For several endpoint-specific analyses, subsets of animals were used according to the sample allocation in the original experimental protocol. Organ indices were assessed in a randomly selected subset of six animals per group, whereas histological analysis, cytokine measurements, qRT-PCR, and 16S rRNA sequencing were performed in randomly selected subsets of three animals per group. Selection of animals for these subset analyses was performed independently of the measured experimental outcomes.

### 2.7. Drug Administration and Dose Setting

Drug administration was performed once daily from day 5 to day 11. The Sanghuang extract doses of 200, 300, and 400 mg/kg/day were selected to provide a graded low-, medium-, and high-dose range with reference to previously reported in vivo doses of *Phellinus*-derived preparations [17]. Accordingly, mice in the SP-L, SP-M, and SP-H groups received Sanghuang extract by oral gavage at 200, 300, and 400 mg/kg/day, respectively. The mesalazine group received mesalazine (Aladdin, Shanghai, China) at 200 mg/kg/day by oral gavage, a dose previously used in mice with DSS-induced colitis [18]. The Caffeic Acid group received caffeic acid at 20 mg/kg/day by oral gavage; this dose was selected with reference to previous oral in vivo studies showing pharmacological activity of caffeic acid at 20 mg/kg in rodents [19]. The 20 mg/kg caffeic acid dose was used as a pharmacological comparator and was not intended to represent a dose equivalent to the caffeic acid content of the Sanghuang extract. The gavage volume was adjusted according to the body weight of each mouse.

### 2.8. Collection and Assessment of Intestinal C. albicans Burden

On day 12, mice were euthanized. The colon, spleen, and thymus were collected. Colon length and weight [20], as well as immune organ weights, were measured, and the following indices were calculated:

Colon edema index = colon weight (g)/body weight (g);

Colon weight-to-length ratio = colon weight (g)/colon length (cm);

Immune organ index = spleen or thymus weight (g)/body weight (g).

Fresh fecal samples were collected on days 1, 3, 5, 7, 9, and 11. Samples were weighed, suspended in sterile PBS, diluted, and 100 µL plated onto selective *C. albicans* chromogenic medium containing penicillin-streptomycin. The inoculated plates were maintained at 37 °C for 48 h, after which the resulting colonies were enumerated, and intestinal *C. albicans* load was calculated for each group at each time point [21]. The biological replicate number for fecal *C. albicans* burden assessment was *n* = 10 mice per group.

### 2.9. Histopathological and Immunohistochemical Analyses of Colon Tissues

Colon tissues collected on day 12 were fixed in formalin for 48 h, thoroughly washed in water, dehydrated using an ethanol gradient, cleared in a 1:1 solution of 50% ethanol and xylene, and subsequently processed for paraffin embedding. Sections with a thickness of 4–7 µm were then prepared from the paraffin blocks.

Hematoxylin and eosin (H&E) staining was carried out after the sections were dewaxed with xylene and passed through decreasing concentrations of ethanol for rehydration. The sections were subsequently stained with hematoxylin, differentiated, rinsed, counterstained with eosin, dehydrated, cleared, and mounted using neutral resin [22].

Periodic acid–Schiff (PAS) staining involved deparaffinization and rehydration, oxidation with 0.5% periodic acid, staining with Schiff reagent in the dark, sequential washing with sodium bisulfite and distilled water, counterstaining with hematoxylin, brief differentiation, rinsing, and mounting [23]. H&E and PAS staining were performed using colon tissues from three independent animals per group (*n* = 3 biological replicates per group). 

ZO-1 immunohistochemical staining was performed on paraffin-embedded colon sections. Colon tissues were fixed in formalin for 48 h, paraffin-embedded, and sectioned at a thickness of 4–7 μm. After deparaffinization and rehydration, antigen retrieval was performed in 0.01 M sodium citrate buffer for 20–30 min under high-pressure heating conditions. Endogenous peroxidase activity was blocked with 3% H_2_O_2_ for 10 min, followed by blocking with normal goat serum at 37 °C for 30 min. Sections were then incubated with the primary antibody against ZO-1 at 4 °C overnight, followed by incubation with an HRP-conjugated secondary antibody (D-3004, Shanghai Changdao Biotechnology Co., Ltd., Shanghai, China) at 37 °C for 60 min. Immunoreactivity was visualized using a DAB staining kit (FL-6001, Shanghai Changdao Biotechnology Co., Ltd., China), followed by hematoxylin counterstaining, dehydration, clearing, and mounting. Images were acquired using an OLYMPUS CX41 microscope equipped with a NIKON D5100 digital camera [24,25]. One animal from each group was examined for ZO-1 immunohistochemistry. Multiple microscopic fields were inspected to assess the staining pattern; however, because only one animal per group was available for this analysis, no inferential statistical comparison was performed. ZO-1 staining was therefore interpreted qualitatively. Representative ZO-1 immunohistochemical images are provided in Supplementary Figure S3.

### 2.10. Measurement of IL-6, IL-1β, and TNF-α Levels in Colon Tissues by ELISA

For ELISA preparation, 100 mg of colon tissue was homogenized on ice with 0.9 mL PBS and subsequently spun at 12,000 rpm for 10 min at 4 °C. The supernatant fraction was carefully collected for further analysis [26,27]. Standard curves were prepared using six concentrations in 96-well plates (50 µL/well), and blank wells contained 50 µL PBS; all standards and blanks were run in triplicate. For each sample well, 10 µL of tissue supernatant was combined with 40 µL of sample diluent. Subsequently, 100 µL of detection antibody was dispensed into all wells except the blank controls, and the plate was maintained at 37 °C for 30 min. After five washing cycles, 50 µL each of chromogenic substrates A and B was introduced, followed by a 15 min incubation in the dark. The enzymatic reaction was terminated by adding stop solution, and the optical density was recorded at 450 nm. Cytokine levels were determined according to the standard curve generated from absorbance values and corresponding concentrations. Three independent biological samples from each group were analyzed (*n* = 3 mice per group).

### 2.11. Total RNA Extraction and RT–qPCR

Approximately 100 mg of colon tissue was processed for total RNA isolation using Trizol reagent (1B14KA7304, Sangon Biotech Co., Ltd., Shanghai, China), followed by dissolution in DEPC-treated water (0000572388, Shanghai Yubo Bio, Shanghai, China). The concentration and purity of RNA were assessed with a NanoDrop 2000 micro-volume spectrophotometer (Thermo Fisher Scientific, Wilmington, DE, USA), and samples with OD_260_/OD_280_ ratios ranging from 1.8 to 2.2 were retained for further experiments. Primers for PI3K, AKT, and mTOR were designed using an online primer design tool (sequences listed in Table 1). cDNA was synthesized using HiFiScript cDNA Synthesis Kit (26323, Kangwei Group, Guangzhou, China), and RT–qPCR was performed with SYBR Green qPCR Mix (102122230407, Beyotime Biotechnology, Shanghai, China) on a StepOnePlus real-time PCR system (Thermo Fisher Scientific). *Actb* was used as the reference gene, and relative mRNA expression levels of *Pik3ca*, *Akt1*, and *Mtor* were calculated using the 2^−ΔΔCt^ method [28,29].

### 2.12. Network Pharmacology Analysis of Sanghuang Extract on C. albicans-Associated UC

Candidate constituents of Sanghuang were collected from the TCMSP database and supplemented by literature mining. A total of 77 candidate constituents were initially collected, of which 62 were retained after in silico screening. Chemical structures were retrieved from PubChem and imported into SwissADME for ADME screening. Compounds with high gastrointestinal absorption (GI absorption = High) and a drug-likeness index ≥2 were retained and subsequently submitted to SwissTargetPrediction for potential target prediction [30,31]. The complete list of the 62 retained candidate constituents and their internal identifiers (SH01–SH62) is provided in Supplementary Table S1. The archived target-prediction dataset contained 6366 compound–target prediction entries, corresponding to 934 unique predicted targets after gene-symbol normalization and de-duplication. The database-derived candidate compounds were used to explore the potential chemical–target space of Sanghuang and should not be interpreted as constituents experimentally confirmed to be present in the administered extract.

Candidate disease-associated targets were retrieved from GeneCards using “ulcerative colitis associated with *Candida albicans*” as an operational search term representing the combined disease context investigated in this study. Because this term does not represent a standardized disease entity, the retrieved targets were treated as candidate disease-associated targets for exploratory network analysis rather than as a definitive UCCA-specific target set. Overlapping targets between Sanghuang-predicted and disease-related targets were identified and used for subsequent analyses. A compound–disease target network was constructed and visualized in Cytoscape (version 3.9.1) [32].

Protein–protein interactions were explored using the STRING database with the organism restricted to *Homo sapiens*. The resulting PPI network was imported into Cytoscape 3.9.1 and analyzed using Network Analyzer, and the top 15 targets ranked by degree were identified as the principal hub targets. Functional annotation and GO and KEGG pathway enrichment analyses were performed using DAVID, with *p* < 0.05 and FDR < 0.01 as the significance thresholds. Enrichment results were visualized using Weishengxin [33]. Among the six constituents detected by UPLC, caffeic acid showed the highest degree centrality in the component–disease network and was therefore selected as a representative candidate for subsequent in vivo comparison and molecular docking.

### 2.13. Molecular Docking

Based on the network-topological analysis described above, caffeic acid was selected for molecular docking to investigate its predicted interactions with AKT1, PIK3CA, mTOR, EGFR, and PTGS2. The experimentally resolved structures of mouse PIK3CA (PDB ID: 4A55, chain A) and PTGS2 (PDB ID: 3NT1, chain A) were obtained from the RCSB Protein Data Bank. Predicted structures were used for the remaining targets. The mouse AKT1 structure was obtained from the AlphaFold Protein Structure Database (UniProt accession P31750; model AF-P31750-F1; global pLDDT = 82.04), whereas a homology model of the mouse EGFR kinase domain (UniProt accession Q01279) was obtained from SWISS-MODEL. The mouse mTOR structure was obtained from the AlphaFold Protein Structure Database (UniProt accession Q9JLN9). Protein sequence, structural-domain, and active-site information was checked against UniProt [34].

Protein structures were prepared by removing water molecules, non-relevant ligands, and other small molecules, followed by addition of polar hydrogen atoms and assignment of Gasteiger charges. AutoDock Tools 1.5.7 was used to generate receptor PDBQT files. The three-dimensional structure of caffeic acid (PubChem CID: 689043) was obtained from PubChem and prepared for docking with charge assignment and rotatable-bond definition before conversion into PDBQT format. Potential ligand-binding pockets were predicted using Cyscore (version 3.10.0), with five candidate pockets generated for each receptor. Molecular docking was performed independently for each candidate pocket using AutoDock Vina 1.2.7 with an exhaustiveness value of 10 and a grid spacing of 0.375 Å. Nine docking poses were generated for each pocket. The pose with the lowest Vina docking score among the five candidate pockets was selected for subsequent analysis. The grid centers and dimensions of the selected docking boxes for each target are provided in Table 2. Docking scores are reported in kcal/mol and were interpreted as computational scores rather than direct evidence of biological binding. The selected docking conformations and intermolecular interactions were visualized using PyMOL 2.6.0. Reference-ligand redocking was additionally performed for the two experimentally resolved protein structures, PIK3CA (PDB ID: 4A55) and PTGS2 (PDB ID: 3NT1), using their corresponding co-crystallized ligands. Redocking was carried out using AutoDock Vina 1.2.7 with the same general docking settings, including an exhaustiveness value of 10 and a grid spacing of 0.375 Å. The redocked poses were superimposed on the experimentally observed ligand conformations, and heavy-atom root-mean-square deviation (RMSD) values were calculated to assess reproduction of the crystallographic binding poses. An RMSD of <2.0 Å was considered indicative of successful pose reproduction. Equivalent reference-ligand redocking was not applicable to AKT1 and mTOR, which were represented by AlphaFold-predicted structures, or to EGFR, which was represented by a homology model.

### 2.14. 16S rRNA Gene Amplicon Sequencing and Bioinformatic Analysis

Fecal samples were collected on day 11 and immediately snap-frozen in liquid nitrogen before storage at −80 °C. Three independent fecal samples from three mice were analyzed per group (*n* = 3 per group; 24 samples in total). Microbial genomic DNA was extracted using the E.Z.N.A.^®^ Stool DNA Kit (Omega Bio-tek, Norcross, GA, USA), and DNA quality was assessed by agarose gel electrophoresis. The V3–V4 hypervariable region of the bacterial 16S rRNA gene was amplified using primers 338F (5′-ACTCCTACGGGAGGCAGCAG-3′) and 806R (5′-GGACTACHVGGGTWTCTAAT-3′) [35]. PCR amplification consisted of an initial denaturation at 95 °C for 5 min, followed by 27 cycles of denaturation at 95 °C for 30 s, annealing at 55 °C for 30 s, and extension at 72 °C for 30 s, with a final extension at 72 °C for 10 min. PCR products were purified, pooled in equimolar amounts, and subjected to paired-end sequencing on an Illumina NextSeq 2000 platform by Shanghai Majorbio Bio-pharm Technology Co., Ltd. (Shanghai, China).

Paired-end reads were quality-filtered using fastp (version 0.23.2) and merged using FLASH (version 1.2.11). The 3′ ends of reads were trimmed using a 10 bp sliding window when the mean quality score within the window was <20. Reads shorter than 50 bp after quality filtering or containing more than five ambiguous bases were discarded. Paired-end reads were merged with a minimum overlap of 10 bp and a maximum mismatch ratio of 0.2 in the overlapping region. Samples were demultiplexed according to barcode and primer sequences, allowing no barcode mismatches and a maximum of two primer mismatches, and sequence orientation was corrected before downstream analysis.

Optimized sequences were dereplicated, singleton sequences were removed, and operational taxonomic units (OTUs) were clustered at 97% sequence similarity using UPARSE (version 7.0.1090), with chimera removal during clustering. All optimized sequences were subsequently mapped to representative OTU sequences at ≥97% similarity to generate the OTU abundance table. Representative OTU sequences were taxonomically classified using the RDP Classifier (version 2.11) against the SILVA Release 138 bacterial 16S rRNA database with a confidence threshold of 0.7. The OTU table was rarefied to 28,227 sequences per sample prior to community-level comparisons.

Alpha diversity was evaluated using the Sobs and Shannon indices, and differences among groups were assessed using the Kruskal–Wallis rank-sum test. Beta diversity was evaluated by principal coordinates analysis (PCoA) based on Bray–Curtis dissimilarity, and overall differences in bacterial community composition among groups were assessed by PERMANOVA (Adonis) with 999 permutations. Genus-level differences among groups were assessed using the Kruskal–Wallis H test with FDR correction for multiple comparisons. The raw 16S rRNA gene sequencing reads generated in this study have been deposited in the Genome Sequence Archive (GSA) at the National Genomics Data Center and are publicly available under accession number CRA051121.

### 2.15. Statistical Analysis

Most graphical representations were prepared using GraphPad Prism version 9.5. Data are presented as mean ± SD unless otherwise specified. Longitudinal body weight data were analyzed using a linear mixed-effects model, with group, time, and the group × time interaction included as fixed effects and individual mouse as a random effect. Dunnett-adjusted post hoc comparisons were used to compare each intervention group with the UCCA model group at individual time points. Fecal *C. albicans* burden was interpreted descriptively because individual-level longitudinal records were not available for repeated-measures reanalysis. For single-time-point outcomes, homogeneity of variance was assessed before analysis. Colon length was analyzed using Welch’s one-way ANOVA followed by Holm-adjusted comparisons with the UCCA model group. Other single-time-point quantitative outcomes were analyzed using ordinary one-way ANOVA followed by Dunnett’s multiple-comparisons test, with the UCCA model group as the reference. A *p* value < 0.05 was considered statistically significant.

## 3. Results

### 3.1. Analysis of Sanghuang Extract

**Figure 1:**
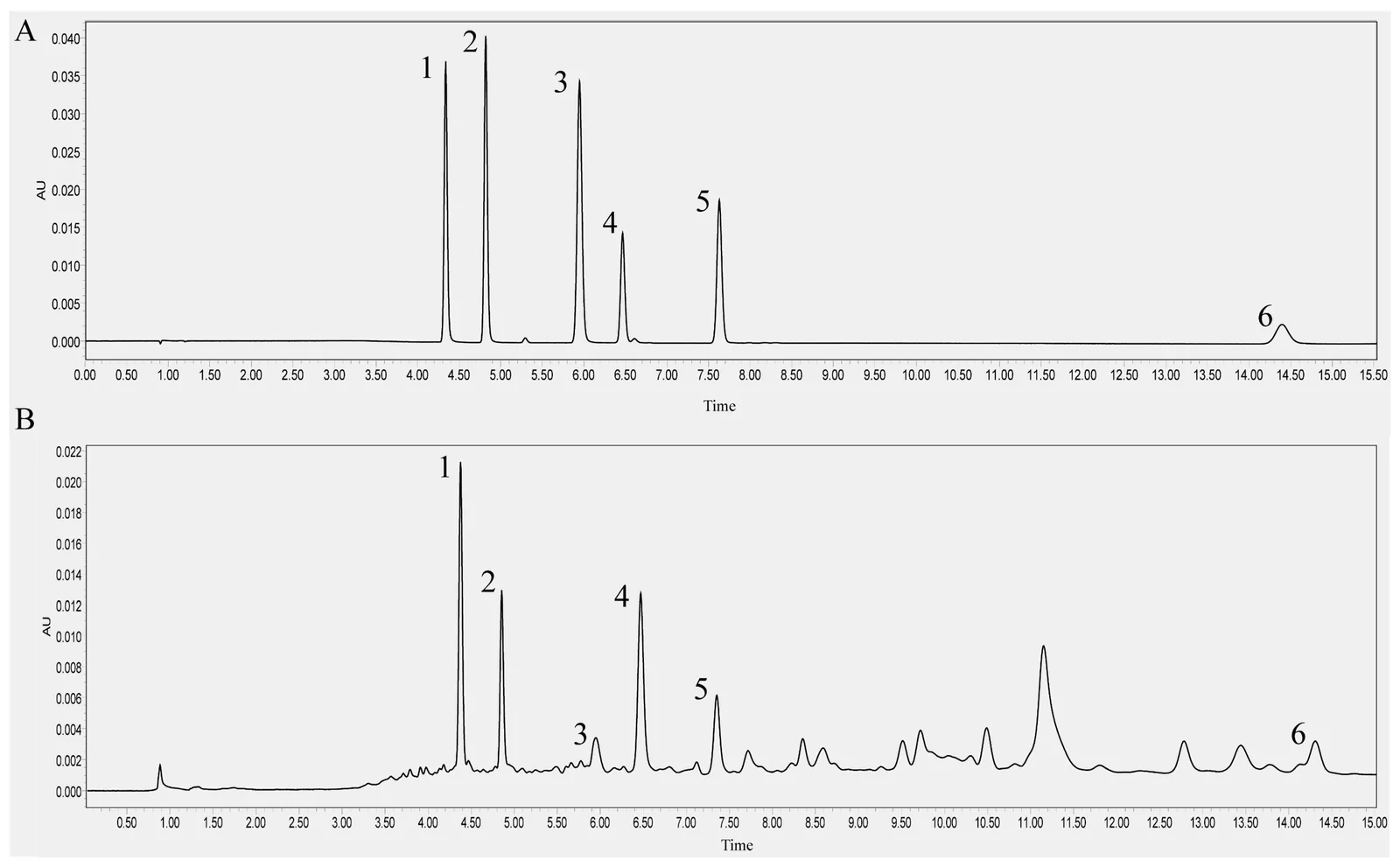
UPLC chromatograms of the mixed standard solution (**A**) and Sanghuang extract (**B**). Peak assignments: (1) protocatechualdehyde; (2) caffeic acid; (3) *p*-coumaric acid; (4) myricitrin; (5) kaempferol-3-*O*-rutinoside; and (6) luteolin.

The chromatograms of the mixed standard solution (Figure 1A) and Sanghuang extract (Figure 1B) were obtained by UPLC. Six chromatographic peaks were assigned by comparison with the corresponding reference standards: Peak 1, protocatechualdehyde; Peak 2, caffeic acid; Peak 3, *p*-coumaric acid; Peak 4, myricitrin; Peak 5, kaempferol-3-*O*-rutinoside; and Peak 6, luteolin. These comparisons supported the qualitative detection of the six constituents in the Sanghuang extract. The optimized extraction procedure showed good reproducibility, with a total polyphenol content of 11.009 ± 0.22 mg GAE/g extract (RSD = 1.96%). Six constituents were qualitatively detected by UPLC based on comparison with reference standards.

### 3.2. Inhibitory Activity of Sanghuang Extract Against C. albicans In Vitro

**Figure 2:**
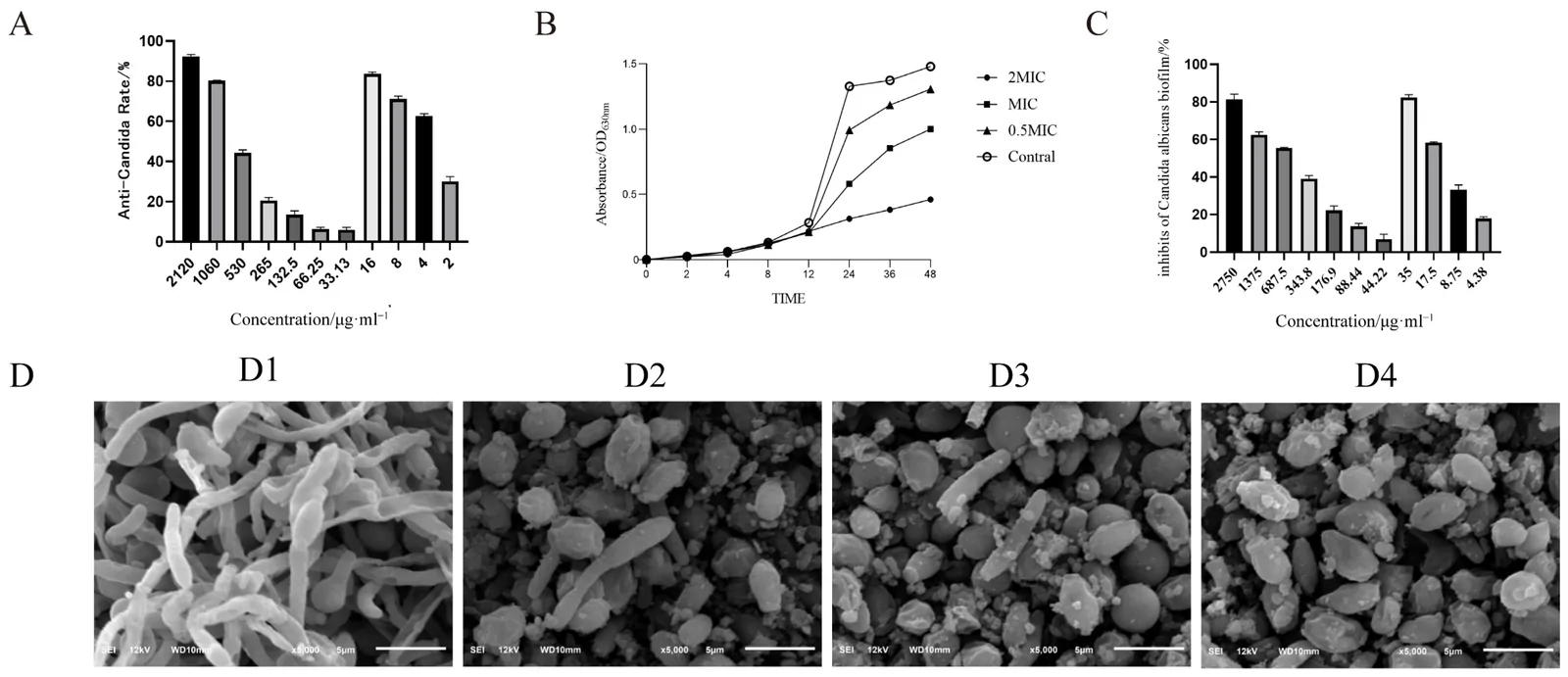
Antifungal and antibiofilm effects of Sanghuang extract against *C. albicans*. (**A**) Anti-*Candida* rate (%) at different concentrations. (**B**) Time–growth curves at 0.5 × MIC, MIC, and 2 × MIC. (**C**) Inhibition of *C. albicans* biofilm formation (%). (**D**) SEM images of *C. albicans* at 12 h: (**D1**), negative control; (**D2**), 0.5 × SMIC_80_; (**D3**), 1 × SMIC_80_; (**D4**), 2 × SMIC_80_.

With increasing concentrations of Sanghuang extract, the inhibitory effect against *C. albicans* was enhanced. The MIC_80_ was 1060 μg/mL, whereas the SMIC_80_ was 2750 μg/mL. Clotrimazole showed MIC_80_ and SMIC_80_ values of 16 μg/mL and 35 μg/mL, respectively (Figure 2A–C). Time–growth curve analysis showed that the extract inhibited fungal growth in a concentration-dependent manner and delayed the logarithmic growth phase at 2120 μg/mL (Figure 2B).

SEM images showed abundant hyphal structures in the untreated control. In Sanghuang extract-treated samples, fewer hyphal structures were observed, together with surface depression and wrinkling of fungal cells. At 2 × SMIC_80_, most observed cells remained in the yeast form, with only limited hyphal structures visible. These observations were qualitative and were not subjected to quantitative morphometric analysis (Figure 2D). The corresponding SEM images obtained at 24 h are provided in Supplementary Figure S2.

### 3.3. Effects of Sanghuang Extract on General Status, Body Weight, and Fecal C. albicans Load in Mice with UC

Mice in the normal group showed glossy fur, normal mental status, and normal food intake, with no hematochezia or obvious colonic edema or hemorrhage. From day 7 onward, mice in the UCCA model group and DSS group showed reduced activity, decreased food and water intake, and unformed feces, with some mice exhibiting hematochezia. Compared with the UCCA model group, these abnormalities were alleviated to varying degrees in all treatment groups, and the Sanghuang extract groups showed a dose-dependent trend. All animals survived until the scheduled endpoint on day 12.

**Figure 3:**
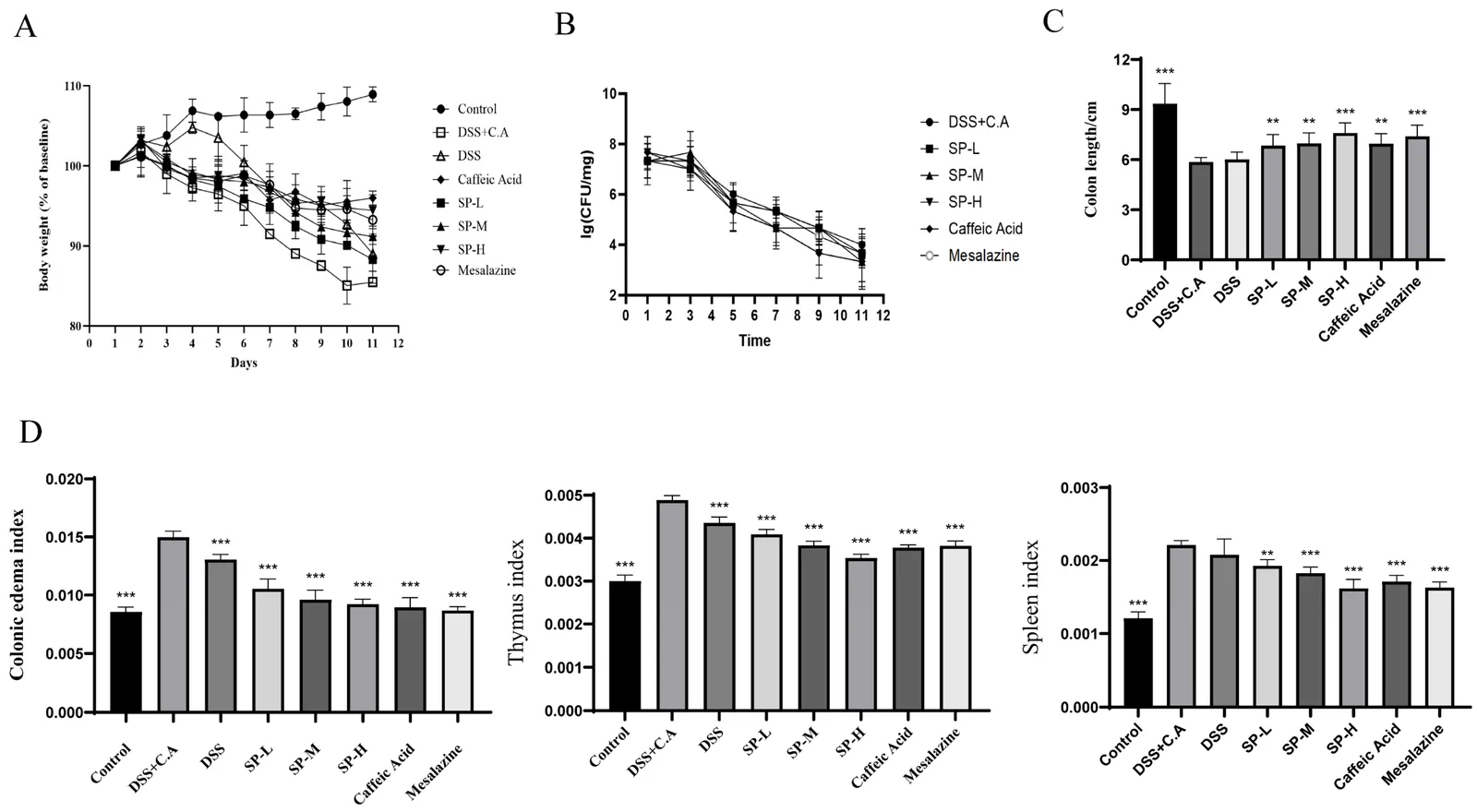
Effects of Sanghuang extract on body weight, fecal *C. albicans* burden, colon length, and organ indices in UCCA mice. (**A**) Relative body weight changes from day 1 to day 11 (*n* = 10 mice per group). Body weight data were analyzed using a linear mixed-effects model with group, time, and group × time interaction as fixed effects and individual mouse as a random effect, followed by Dunnett-adjusted comparisons with the UCCA model group at individual time points. (**B**) Fecal *C. albicans* burden (log CFU/mg) over time (*n* = 10 mice per group). These data were interpreted descriptively and were not subjected to inferential statistical analysis because individual-level longitudinal records were unavailable for repeated-measures reanalysis. (**C**) Representative images and quantitative analysis of colon length (*n* = 10 biological replicates per group). Colon-length data were analyzed using Welch’s one-way ANOVA followed by Holm-adjusted comparisons with the UCCA model group. (**D**) Colonic edema index, thymus index, and spleen index (*n* = 6 biological replicates per group). These outcomes were analyzed using ordinary one-way ANOVA followed by Dunnett’s multiple-comparisons test, with the UCCA model group as the reference. Data are presented as mean ± SD where applicable. ** *p* < 0.01, *** *p* < 0.001, versus the UCCA model group. Control, blank control group; DSS + C.A, UCCA model group; DSS, DSS-only group; SP-L, low-dose Sanghuang extract group; SP-M, medium-dose Sanghuang extract group; SP-H, high-dose Sanghuang extract group. UCCA refers to ulcerative colitis accompanied by intestinal *C. albicans* colonization.

Body weight was expressed as a percentage of the day 1 baseline value for each animal. A linear mixed-effects model incorporating group, time, and the group × time interaction showed a significant effect of time and a significant group × time interaction (both *p* < 0.001). Dunnett-adjusted comparisons with the UCCA model group showed significant differences at selected later time points: SP-M on days 7, 10, and 11; SP-H on days 8–11; caffeic acid on days 8–11; and mesalazine on days 7–11 (all adjusted *p* < 0.05). No significant difference between SP-L and the UCCA model group was detected at any individual time point (Figure 3A).

Fecal *C. albicans* burden was monitored on days 1, 3, 5, 7, 9, and 11. The group-level data showed decreasing numerical trends in several intervention groups during the later stage of the experiment. However, because individual-level longitudinal records were not available for repeated-measures reanalysis, no inferential statistical comparison was performed for this outcome, and the findings were interpreted descriptively (Figure 3B).

### 3.4. Effects of Sanghuang Extract on Colon Damage and Inflammation in UCCA Mice

Colon length differed significantly among the experimental groups (Welch’s one-way ANOVA, *p* < 0.001). Holm-adjusted comparisons using the UCCA model group as the reference showed significantly greater colon length in the SP-L, SP-M, SP-H, caffeic acid, and mesalazine groups (all adjusted *p* < 0.01), whereas the DSS-only group did not differ significantly from the UCCA model group (Figure 3C). The colonic edema index and thymus index were significantly lower in all intervention groups than in the UCCA model group (all *p* < 0.001). The spleen index was also significantly lower in the SP-L group (*p* < 0.01) and in the SP-M, SP-H, caffeic acid, and mesalazine groups (all *p* < 0.001) compared with the UCCA model group (Figure 3D).

**Figure 4:**
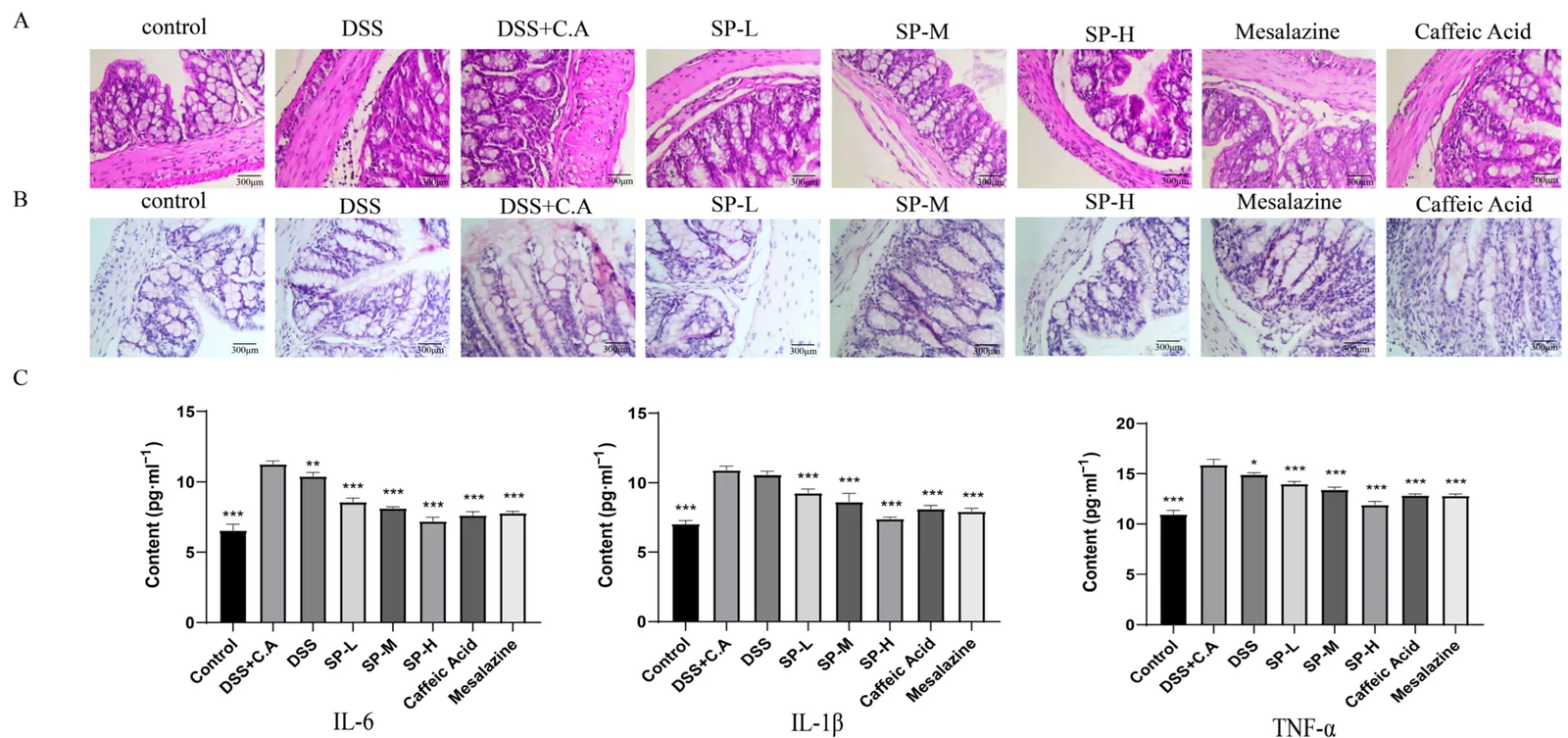
Effects of Sanghuang extract on colonic histopathology, ZO-1 immunostaining, and inflammatory responses in UCCA mice. (**A**) Representative H&E-stained colon sections; (**B**) representative PAS-stained colon sections showing fungal structures and (**C**) colonic levels of IL-6, IL-1β, and TNF-α. H&E and PAS staining was performed in three independent animals per group (*n* = 3 biological replicates per group), and representative sections are shown. These observations were interpreted qualitatively because no formal histological scoring or inferential statistical analysis was performed. Cytokine data are presented as mean ± SD (*n* = 3 mice per group) and were analyzed using one-way ANOVA followed by Dunnett’s multiple-comparisons test, with the UCCA model group as the reference. * *p* < 0.05, ** *p* < 0.01, *** *p* < 0.001 versus the UCCA model group. Original magnification: 200×.

Histopathological examination showed an intact colonic mucosal structure, regularly arranged glands, and abundant goblet cells in the Control group. In contrast, representative sections from the DSS and UCCA model groups showed mucosal erosion, congestion and edema, crypt abnormalities, goblet-cell loss, and epithelial damage. Representative sections from the Sanghuang extract-treated groups showed less severe histopathological alterations than those observed in the UCCA model group. Similar qualitative improvements were observed in the caffeic acid and mesalazine groups (Figure 4A). These observations were descriptive and were not based on a formal histological scoring system.

PAS staining showed hyphae and/or spore-like fungal structures in the colonic mucosa of the UCCA model group. Fewer fungal structures were visually observed in representative sections from the Sanghuang extract-treated groups, as well as in the caffeic acid and mesalazine groups (Figure 4B). These findings were interpreted qualitatively.

ZO-1 immunohistochemical staining showed differences in staining intensity and distribution among the experimental groups (Supplementary Figure S3). Because only one animal per group was available for ZO-1 immunohistochemical analysis, no inferential statistical comparison was performed, and the staining patterns were interpreted qualitatively.

Colonic IL-6, IL-1β, and TNF-α levels differed significantly among the experimental groups (all one-way ANOVA, *p* < 0.0001). Compared with the UCCA model group, IL-6 levels were significantly lower in the SP-L, SP-M, SP-H, caffeic acid, and mesalazine groups (all Dunnett-adjusted *p* < 0.001). Similar reductions were observed for IL-1β and TNF-α in all intervention groups (all adjusted *p* < 0.0001). These findings indicate lower colonic pro-inflammatory cytokine levels following the interventions (Figure 4C).

### 3.5. Effects of Sanghuang Extract on the Intestinal Bacterial Community in UCCA Mice

A total of 1,228,968 quality-filtered sequences were obtained from the 24 fecal samples, with 40,173–65,015 sequences per sample (mean, 51,207 sequences per sample). For downstream community analyses, the OTU table was rarefied to 28,227 sequences per sample. At the OTU level, neither observed richness (Sobs) nor Shannon diversity differed significantly among the eight experimental groups (Kruskal–Wallis test: Sobs, H = 6.40, *p* = 0.494; Shannon, H = 7.92, *p* = 0.340). Thus, the present data did not demonstrate significant group-level differences in alpha diversity (Figure S1A,B).

PCoA based on Bray–Curtis dissimilarity showed variation in bacterial community composition among the experimental groups. PC1 and PC2 explained 31.84% and 17.68% of the total variation, respectively. PERMANOVA indicated an overall difference in bacterial community composition among the eight groups (F = 1.781, R^2^ = 0.438, *p* = 0.011; Figure S1C).

**Figure 5:**
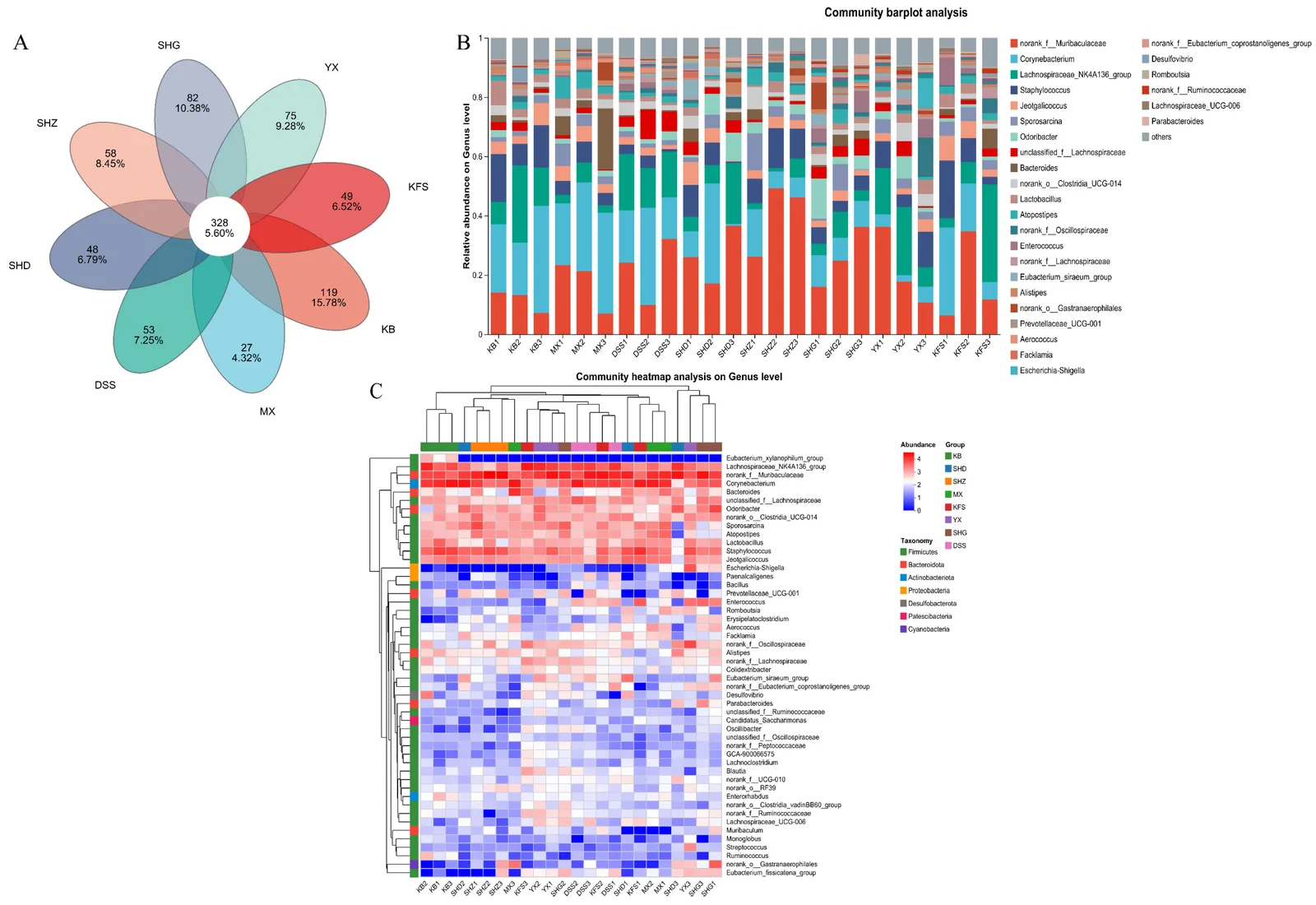
Intestinal bacterial community composition in UCCA mice. (**A**) OTU Venn diagram showing shared and unique OTUs among groups. (**B**) Relative abundance of genera across all samples. (**C**) Heatmap of genus-level composition showing differences among groups. The group codes used in the microbiota plots correspond to the experimental groups as follows: KB, Control; MX, DSS + C.A (UCCA model); DSS, DSS-only group; KS, Caffeic Acid; SHD, SP-L; SHZ, SP-M; SHG, SP-H; and YX, mesalazine.

**Figure 6:**
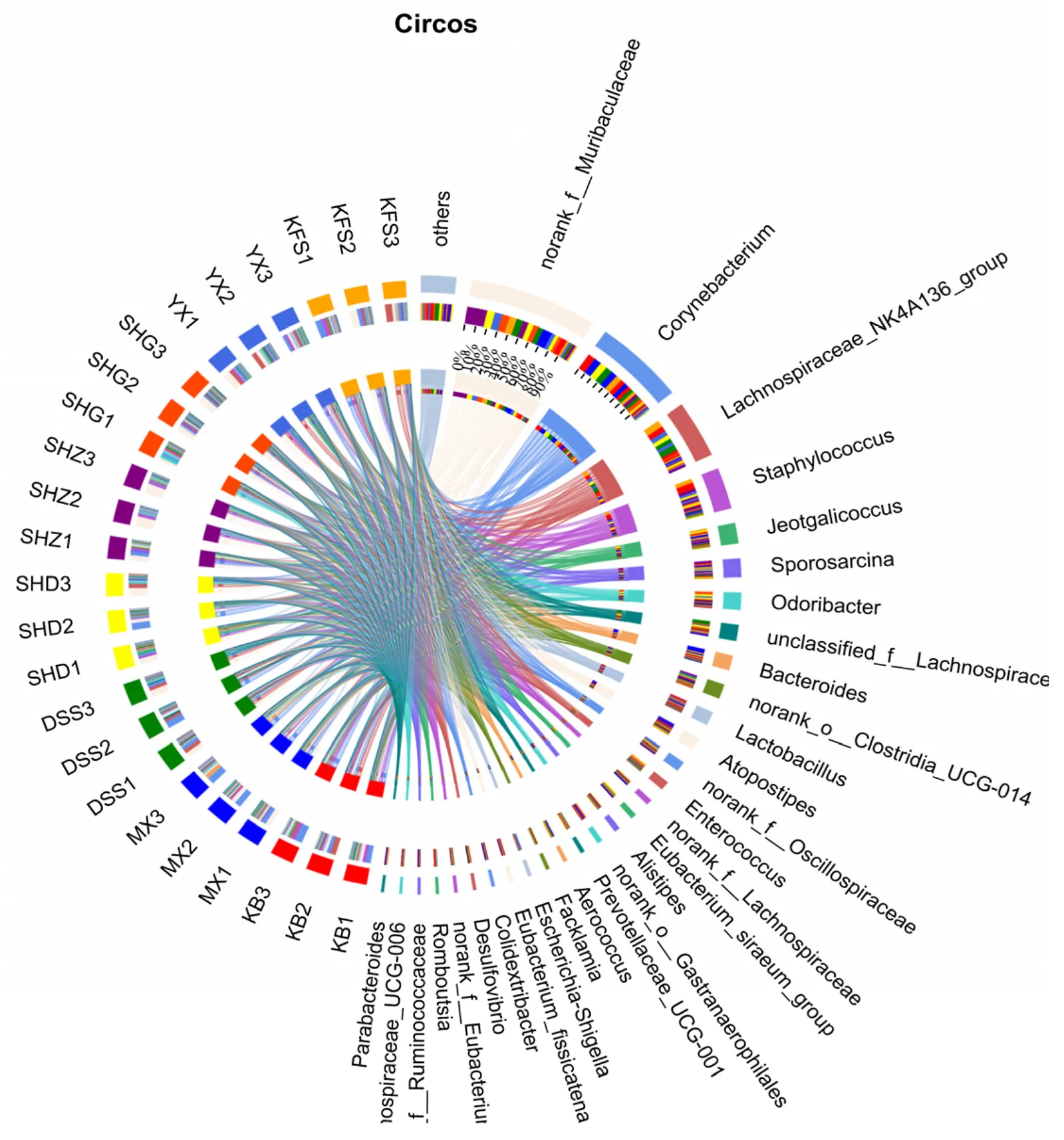
Circos plot showing the relationships between experimental groups and dominant bacterial genera.

At the genus level, descriptive differences in bacterial community composition were observed among groups. Cluster analysis and Circos plots further illustrated differences in community profiles among the experimental groups. (Figure 5B,C and Figure 6).

Several genera showed nominal differences among groups in the Kruskal–Wallis analysis; however, none remained statistically significant after FDR correction. Therefore, these genus-level observations were interpreted as exploratory compositional trends rather than confirmed differential taxa.

### 3.6. Network Pharmacology Prediction of Potential Targets and Pathways Associated with Sanghuang

A total of 77 candidate Sanghuang constituents were collected from the TCMSP database and the literature, of which 62 were retained after in silico screening (Supplementary Table S1). These database-derived candidates represented a broader predicted chemical space of Sanghuang, whereas only six constituents were experimentally detected in the present extract by UPLC. The archived SwissTargetPrediction dataset contained 6366 compound–target prediction entries, corresponding to 934 unique predicted targets after gene-symbol normalization and de-duplication. The six constituents detected by UPLC corresponded to 285 predicted targets, accounting for 30.51% of the total Sanghuang-related target set. In addition, 573 candidate disease-associated targets were retrieved from GeneCards and retained after de-duplication. Venn analysis identified 112 overlapping targets between the Sanghuang-related target set and the candidate disease-associated target set, of which 45 were associated with the six UPLC-detected constituents. Topological analysis of the component–disease network showed that caffeic acid had the highest degree value among the six UPLC-detected constituents (Degree = 26). Therefore, caffeic acid was selected as a representative candidate constituent for subsequent in vivo comparison and molecular docking.

**Figure 7:**
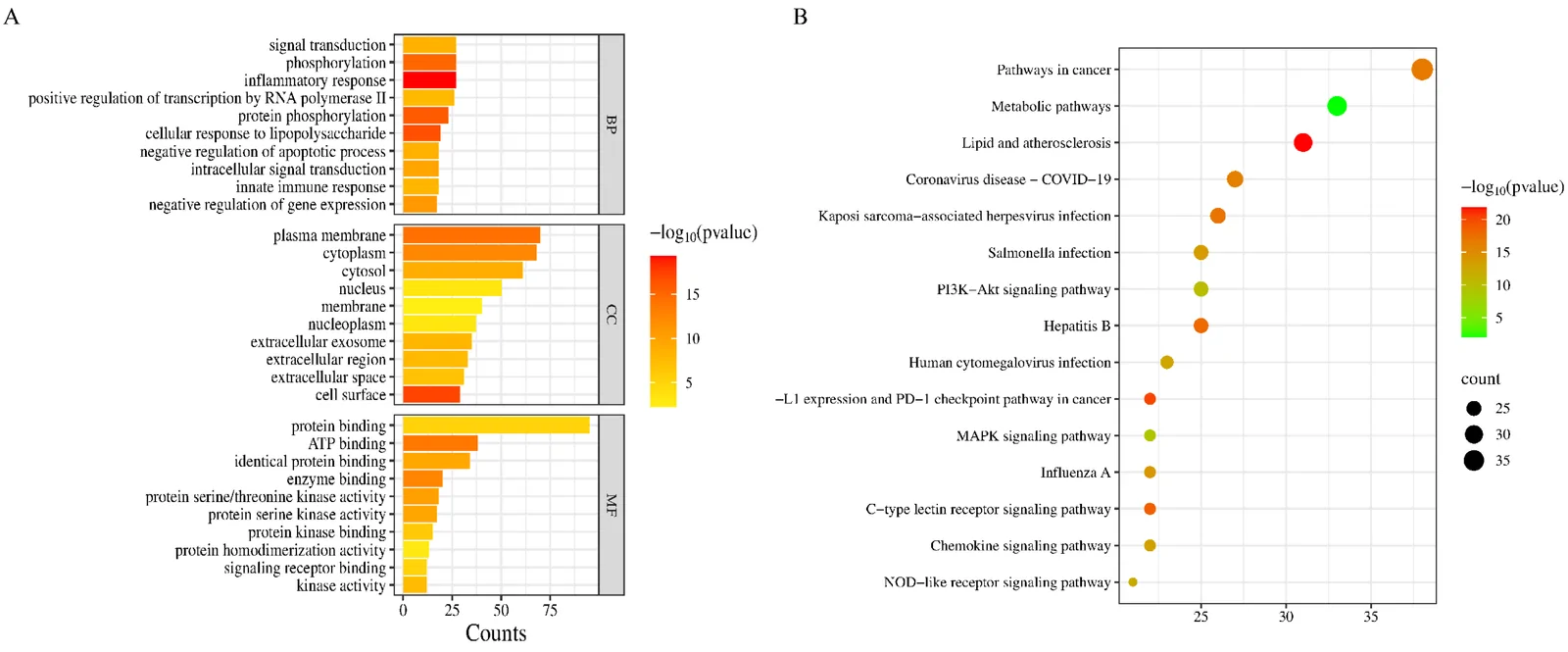
Functional enrichment analysis of Sanghuang extract-related targets. (**A**) GO enrichment analysis of overlapping targets. (**B**) KEGG pathway enrichment analysis of overlapping targets.

**Figure 8:**
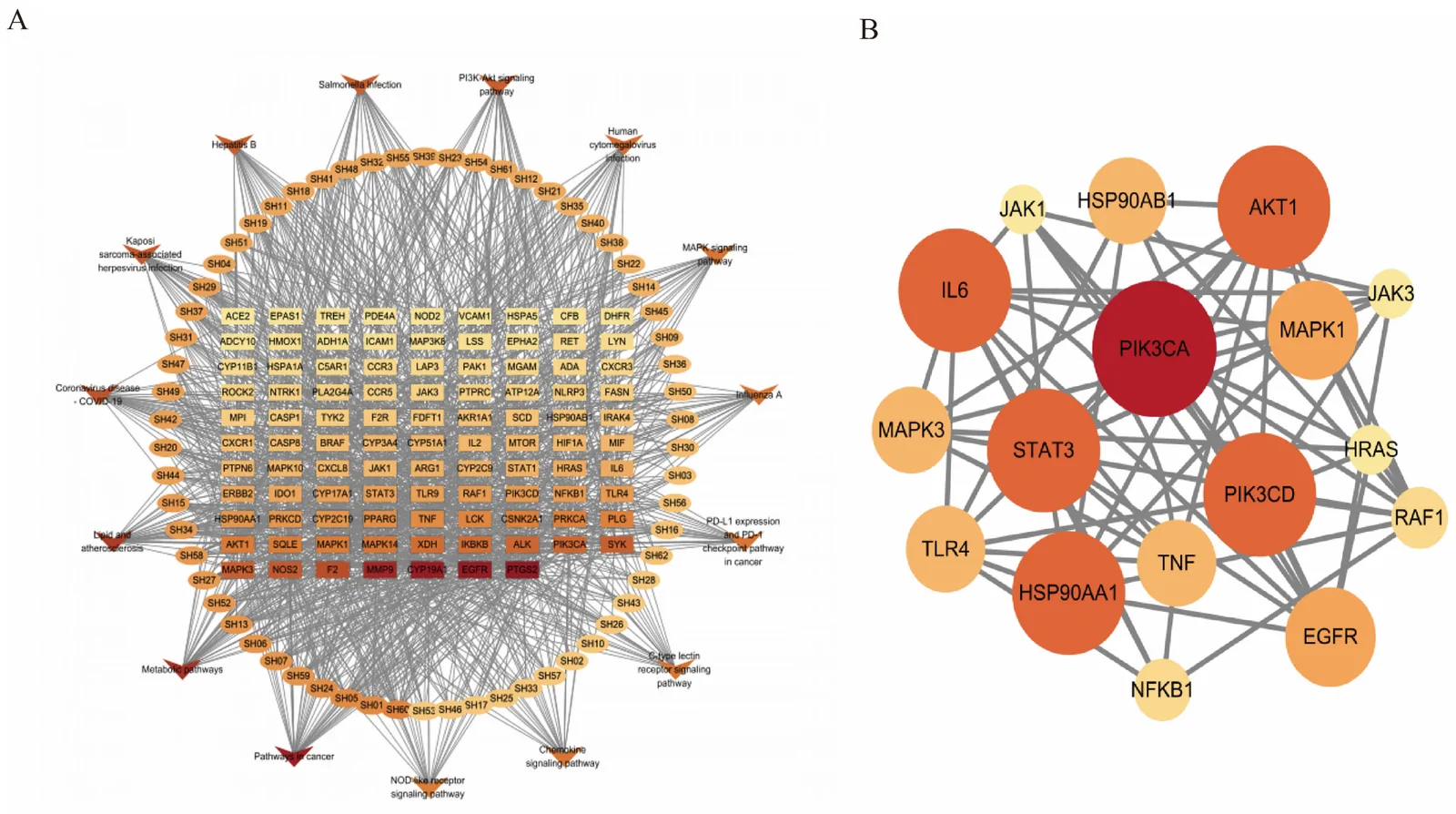
Component–disease–pathway network and PPI analysis of Sanghuang-related predicted targets. (**A**) Network of database-derived candidate constituents, candidate disease-associated targets, and related signaling pathways. (**B**) protein–protein interaction (PPI) network highlighting highly connected predicted targets.

GO functional enrichment showed that these common targets were mainly associated with the regulation of gene expression, cellular signaling, inflammatory processes, and molecular binding activity (Figure 7A). KEGG analysis further highlighted PI3K-AKT, MAPK, and PD-L1 signaling as key enriched pathways. (Figure 7B). A component–disease–pathway network was further constructed (Figure 8A), and PPI analysis identified important targets including PTGS2, EGFR, CYP19A1, PIK3CA, AKT1, and PIK3CD (Figure 8B).

### 3.7. Molecular Docking Analysis of Caffeic Acid with Selected Targets

**Figure 9:**
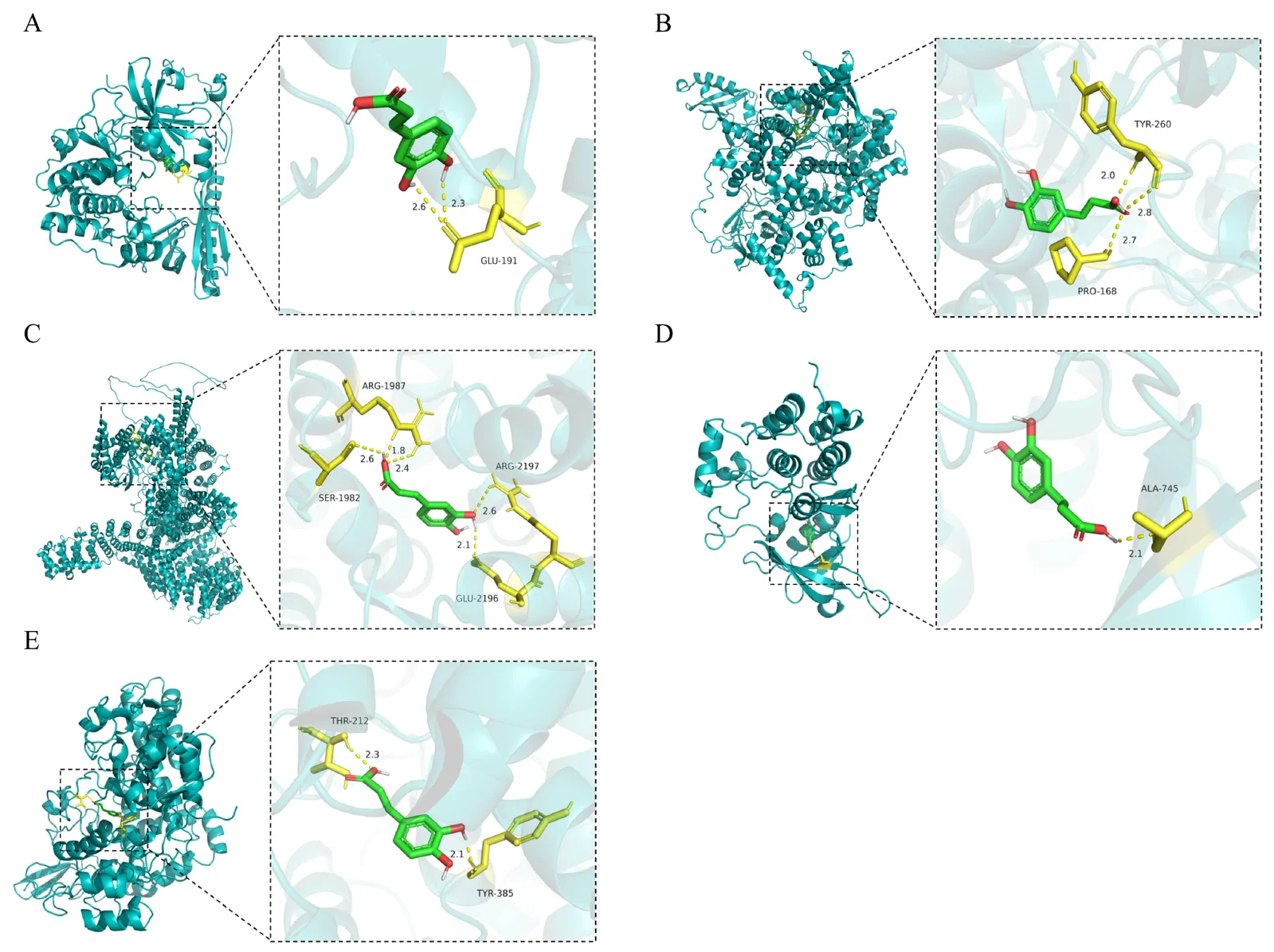
Molecular docking analysis of caffeic acid with selected target proteins. Predicted docking binding conformations of caffeic acid with (**A**) AKT1, (**B**) PIK3CA, (**C**) mTOR, (**D**) EGFR, and (**E**) PTGS2. The protein structures are shown in cyan, caffeic acid in green, and the interacting amino acid residues in yellow.

Molecular docking predicted potential interactions of caffeic acid with AKT1, PIK3CA, mTOR, EGFR, and PTGS2 (Figure 9A–E). The lowest Vina docking scores for caffeic acid with AKT1, PIK3CA, mTOR, EGFR, and PTGS2 were −6.228, −7.438, −6.864, −6.344, and −6.659 kcal/mol, respectively. Predicted hydrogen-bond interactions were observed with GLU-191 in AKT1; TYR-260 and PRO-168 in PIK3CA; SER-1982, ARG-1987, GLU-2196, and ARG-2197 in mTOR; ALA-745 in EGFR; and THR-212 and TYR-385 in PTGS2. Among the five evaluated targets, PIK3CA yielded the lowest Vina docking score (−7.438 kcal/mol). However, the PIK3CA reference ligand did not reproduce the crystallographic binding pose within the predefined 2.0 Å RMSD criterion during redocking validation. Therefore, the lower docking score obtained for PIK3CA should not be interpreted as evidence of stronger or validated binding. These validation results further support a cautious interpretation of the docking predictions, particularly for PIK3CA and for targets represented by predicted or homology-modeled structures.

### 3.8. Effects of Sanghuang Extract on PI3K/AKT/mTOR Pathway-Related Gene Expression

**Figure 10:**
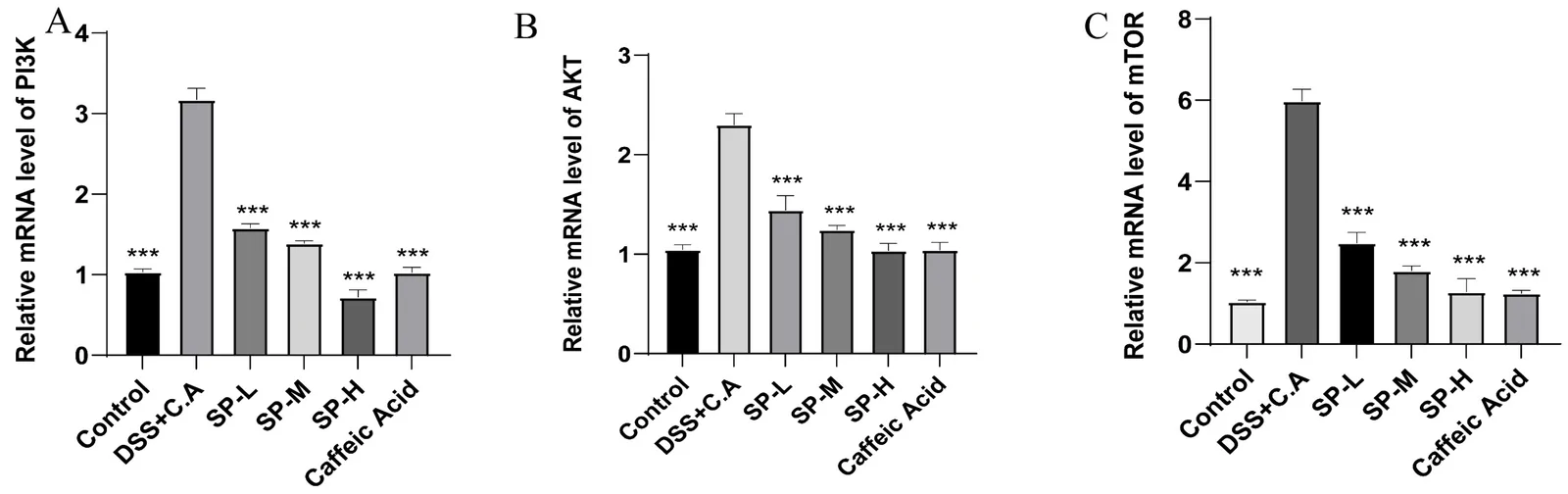
Effects of Sanghuang extract on the mRNA expression of PI3K/AKT/mTOR pathway-related genes in colon tissue. mesalazine was not included in the qRT-PCR analysis: (**A**) relative mRNA expression of *Pik3ca*; (**B**) relative mRNA expression of *Akt1*; (**C**) relative mRNA expression of *Mtor*. Data are presented as mean ± SD (*n* = 3 biological replicates per group). Differences among groups were analyzed using ordinary one-way ANOVA followed by Dunnett’s multiple-comparisons test, with the UCCA model group as the reference. *** *p* < 0.001 versus the UCCA model group.

To examine whether Sanghuang treatment was associated with changes in PI3K/AKT/mTOR pathway-related gene expression, qRT-PCR was used to determine the mRNA expression levels of *Pik3ca*, *Akt1*, and *Mtor* in colon tissues. Compared with the UCCA model group, the mRNA expression levels of *Pik3ca*, *Akt1*, and *Mtor* were significantly lower in the SP-L, SP-M, SP-H, and caffeic acid groups (all Dunnett-adjusted *p* < 0.001; Figure 10). mesalazine was not included in the qRT-PCR analysis. These findings indicate that Sanghuang treatment was associated with reduced mRNA expression of PI3K/AKT/mTOR pathway-related genes in colon tissue.

## 4. Discussion

*C. albicans* commonly inhabits the gastrointestinal tract as part of the intestinal fungal community, but it can acquire pathogenic features when the intestinal microenvironment is disturbed [36]. Loss of microbial balance together with mucosal barrier injury may favor fungal overgrowth and virulence-associated changes, including biofilm development, hyphal transition, and activation of mucosal inflammatory responses, thereby worsening colonic inflammation. In the present study, Sanghuang extract showed inhibitory activity against *C. albicans* in vitro and was associated with reduced colitis-associated inflammatory changes in mice with *C. albicans* colonization. The in vitro findings indicated that Sanghuang extract affected several *C. albicans*-associated phenotypes, including fungal growth, biofilm formation, and hyphal development. In mice with UCCA, Sanghuang extract treatment was associated with reduced colitis-associated inflammatory changes, descriptive changes in fecal *C. albicans* burden, and alterations in intestinal bacterial community composition. Previous studies have already reported in vitro and in vivo inhibitory effects of Sanghuang mushroom extracts against *C. albicans* [37]. Therefore, the novelty of the present study does not lie simply in demonstrating the antifungal activity of Sanghuang extract. In our previous work, we investigated cinnamon essential oil in a related DSS/*C. albicans* model [38]. Although the previous and present studies share a similar disease-model framework, they differ in the intervention and mechanistic focus. In contrast to these previous studies, the present work evaluates Sanghuang extract in the DSS/*C. albicans* disease context while integrating qualitative UPLC characterization, antifungal phenotypic assays, assessment of colonic inflammation and intestinal bacterial communities, network pharmacology, molecular docking, and qRT-PCR analysis of PI3K/AKT/mTOR pathway-related gene expression. Among the six constituents qualitatively detected by UPLC, caffeic acid showed the highest network connectivity in the component–disease analysis and was therefore selected as a representative candidate constituent for further in vivo comparison and molecular docking. This selection was based on network topology rather than evidence that caffeic acid was the most abundant or pharmacologically most active constituent of the extract. Because the caffeic acid content of the Sanghuang extract was not quantitatively determined, the 20 mg/kg caffeic acid group should be interpreted as a pharmacological comparator rather than a dose-equivalent comparator to the Sanghuang extract. Thus, the present study extends previous Sanghuang/*Candida* and DSS/*C. albicans* studies by examining fungal colonization, host inflammatory responses, intestinal bacterial community changes, and predicted molecular targets within the same experimental framework. These findings suggest that the effects of Sanghuang extract in this model were associated with antifungal, anti-inflammatory, and intestinal bacterial community-related changes.

The in vitro results showed that Sanghuang extract had definite inhibitory activity against *C. albicans*, although its antifungal potency was weaker than that of the positive control drug. Importantly, Sanghuang extract not only inhibited fungal growth in a concentration-dependent manner, but also suppressed biofilm formation and hyphal development. These two virulence-related phenotypes are closely associated with fungal adhesion, persistence, and tissue invasion [39]. Scanning electron microscopy qualitatively showed fewer hyphal structures and visible surface alterations, including depression and wrinkling, in Sanghuang extract-treated cells compared with the untreated control. These qualitative observations are consistent with an effect of Sanghuang extract on fungal morphology, although quantitative assessment of hyphal development was not performed.

The animal experiments further showed that Sanghuang extract was associated with improvements in several disease-related outcomes in UCCA mice [37]. Body weight trajectories differed between several intervention groups and the UCCA model group at selected later time points, whereas fecal *C. albicans* burden showed descriptive decreasing trends in several intervention groups. Because fecal *C. albicans* burden also declined over time in the untreated UCCA group, the numerical decreases observed in the intervention groups should not be interpreted as evidence of a treatment-specific antifungal effect in vivo. Colon length, colonic edema, and immune-organ indices differed significantly between the intervention groups and the UCCA model group. Representative histological sections showed less severe mucosal alterations in the Sanghuang extract-treated groups than in the UCCA model group, while PAS staining suggested fewer fungal structures in the colonic mucosa. ZO-1 immunostaining was evaluated qualitatively because only one animal per group was available for this analysis. Therefore, the observed differences in staining intensity and distribution should be regarded as descriptive and do not permit conclusions regarding changes in epithelial barrier function. Quantitative ELISA analysis showed significantly lower colonic IL-6, IL-1β, and TNF-α levels in the Sanghuang extract-treated groups compared with the UCCA model group. Taken together, these findings indicate that Sanghuang extract treatment was associated with descriptive reductions in fecal *C. albicans* burden and with reduced colitis-associated inflammatory changes in this model.

Alterations in intestinal bacterial community composition have been reported in UC and may vary with disease status [40]. In the present study, alpha-diversity analysis did not identify significant differences in Sobs or Shannon diversity among the experimental groups, whereas beta-diversity analysis showed an overall difference in bacterial community composition. At the phylum and genus levels, descriptive differences in relative abundance were observed among groups. However, although several genera showed nominal differences in the Kruskal–Wallis analysis, none remained statistically significant after FDR correction. Therefore, these taxonomic patterns should be interpreted as exploratory compositional trends rather than confirmed treatment-specific changes in individual bacterial taxa. Moreover, because 16S rRNA gene sequencing characterizes bacterial communities rather than the intestinal mycobiota, the present findings do not establish restoration of the overall intestinal bacterial community or a causal microbiota-mediated mechanism.

Network pharmacology analysis provided exploratory clues regarding potential molecular mechanisms associated with the disease context examined in this study. Several overlapping predicted targets were identified between Sanghuang-related targets and the candidate disease-associated target set. Enrichment analysis indicated that these overlapping targets were mainly associated with inflammation-related processes, cellular signaling, and molecular binding functions. Pathway enrichment further highlighted PI3K-AKT signaling, together with MAPK- and PD-L1-associated signaling pathways, as potentially relevant pathways [41,42]. It should be noted that the network pharmacology analysis was based on a broader set of database-derived candidate constituents, whereas only six constituents were experimentally detected in the administered Sanghuang extract by UPLC. Therefore, the network-derived targets and pathways should be interpreted as hypothesis-generating predictions rather than direct evidence that all database-derived compounds were present in or contributed to the activity of the extract. qRT-PCR results showed that treatment markedly reduced the colonic mRNA levels of *Pik3ca*, *Akt1*, and *Mtor*. Combined with the observed reductions in inflammatory cytokines, these findings indicate an association between Sanghuang treatment and reduced expression of PI3K/AKT/mTOR pathway-related genes. However, changes in mRNA abundance alone do not demonstrate inhibition of PI3K/AKT/mTOR pathway activation. Assessment of pathway activity would require protein-level and phosphorylation analyses.

Molecular docking was further conducted to explore the potential contribution of caffeic acid, one of the constituents identified in Sanghuang extract, to the predicted molecular mechanisms. Molecular docking predicted possible interactions of caffeic acid with PTGS2, EGFR, PIK3CA, AKT1, and mTOR, with PIK3CA yielding the lowest Vina docking score among the five evaluated targets. However, because the PIK3CA redocking procedure did not reproduce the crystallographic pose within the predefined RMSD criterion, this score should not be interpreted as evidence of stronger or validated binding. These computational results provide hypothesis-generating structural information regarding possible interactions of caffeic acid with inflammation- and PI3K/AKT/mTOR-related proteins. Together with the network pharmacology results and the altered expression of PI3K/AKT/mTOR pathway-related genes observed in colon tissues, the docking analysis supports further investigation of these candidate molecular interactions. Nevertheless, molecular docking scores do not demonstrate direct molecular binding or target inhibition, which would require biochemical or biophysical validation. Reference-ligand redocking provided partial validation of the docking workflow. The PTGS2–ligand complex reproduced the crystallographic binding pose with an RMSD of 0.33 Å, whereas the PIK3CA reference ligand did not reproduce the experimental pose within the 2.0 Å criterion. Moreover, equivalent crystallographic redocking validation was not applicable to AKT1 and mTOR, which were represented by AlphaFold-predicted structures, or to the homology-modeled EGFR structure. Therefore, the docking results should still be regarded as exploratory computational predictions rather than evidence of direct molecular binding or target inhibition.

Certain limitations should be taken into consideration when interpreting the findings of this study. First, Sanghuang extract contains multiple constituents, and although six constituents were qualitatively detected by UPLC, their absolute concentrations were not determined using a validated quantitative analytical method. Therefore, the relative contribution of individual constituents and their possible synergistic interactions cannot be established from the present chemical analysis. Although the 20 mg/kg caffeic acid dose was selected based on previous literature, caffeic acid was not quantitatively determined in the administered Sanghuang extract. Therefore, the caffeic acid group should be interpreted as a pharmacological comparator rather than as a dose-equivalent comparison with the Sanghuang extract. Second, the mechanistic validation in this study focused mainly on pathway-related gene expression, and further confirmation at the protein and functional levels is still needed. Third, although alterations in intestinal bacterial community composition were observed, the causal relationships between specific bacterial taxa and the intervention effect of Sanghuang extract remain to be clarified. Fourth, the antifungal experiments were performed using only one *C. albicans* strain (SN250), and the in vivo findings were obtained from an acute murine model; therefore, the generalizability of these findings to other *C. albicans* strains and disease settings remains limited. Fifth, the study did not include a Sanghuang-treated DSS-only group. Therefore, the present experimental design cannot fully distinguish the direct anti-inflammatory effects of Sanghuang extract from effects that may arise secondarily from reduced *C. albicans* colonization. Sixth, some endpoint-specific analyses were performed using relatively small biological subsets and may limit statistical precision. Future studies should consider incorporate endpoint-specific power analyses and sample-size calculations during the experimental design stage. Future studies should therefore further characterize the bioactive constituents of Sanghuang extract, evaluate additional *C. albicans* strains and disease models, validate pathway-related changes at the protein and functional levels, and further clarify the relationships among Sanghuang extract, fungal colonization, host inflammatory responses, and intestinal bacterial communities.

## 5. Conclusions

Sanghuang extract exhibited inhibitory activity against *C. albicans* growth and biofilm formation in vitro. In a mouse model of UC with *C. albicans* colonization, Sanghuang extract alleviated colonic injury and decreased inflammatory cytokine levels, with descriptive reductions in fecal *C. albicans* burden and changes in intestinal bacterial community composition. Moreover, Sanghuang treatment was associated with reduced colonic mRNA expression of PI3K, AKT, and mTOR.

## Figures and Tables

**Table 1:** Primer sequences for PI3K/AKT/mTOR pathway-related genes.

Primer Name	Sequences (5′ to 3′)	Number of Bases
*Pik3ca*-F	CTGCAGTTCAACAGCCACAC	20
*Pik3ca*-R	ATGCTGCTTGATGGTGTGGA	20
*Akt1*-F	ATGAACGACGTAGCCATTGTG	21
*Akt1*-R	TTGTAGCCAATAAAGGTGCCAT	22
*Mtor*-F	ACTGTGTCTTGGCATCCCTG	20
*Mtor*-R	GGTTCATGCTGCTTAGTCGG	20
*Actb*-F	ACTGTCGAGTCGCGTCC	17
*Actb*-R	CTGACCCATTCCCACCATCA	20

**Table 2:** Molecular docking parameters and Vina docking scores for caffeic acid with the selected target proteins.

Target	Selected Pocket	Grid Center (X, Y, Z)	Grid Size (X, Y, Z), Å	Lowest Vina Docking Score (kcal/mol)
AKT1	4	−9.751, 9.972, −10.995	19, 19, 19	−6.228
PIK3CA	1	−11.047, −33.267, 48.727	29, 33, 31	−7.438
mTOR	2	28.541, 17.401, −32.248	19, 19, 29	−6.864
EGFR	1	−34.833, 43.036, 24.018	26, 29, 32	−6.344
PTGS2	2	−27.674, −36.167, −19.065	19, 26, 19	−6.659

## Data Availability

The raw 16S rRNA gene sequencing reads generated in this study have been deposited in the Genome Sequence Archive (GSA) at the National Genomics Data Center and are publicly available under accession number CRA051121. Other raw data supporting the findings of this study are available from the corresponding author upon reasonable request because some of the data are intended to support a patent application.

## Supplementary Materials

The following supporting information can be downloaded at: https://www.mdpi.com/article/10.3390/pathogens15101071/s1, Figure S1: Alpha- and beta-diversity analyses of the intestinal bacterial community. Figure S2: Representative SEM images of *C. albicans* after 24 h exposure to Sanghuang extract. Table S1: Candidate constituents and PubChem identifiers used in the network pharmacology analysis. Figure S3: Representative ZO-1 immunohistochemical staining of colon tissues from the experimental groups.
