# Supplementary material for: Effects of Sanghuang Extract on Candida albicans Colonization and Intestinal Inflammation in a DSS-Induced Colitis Mouse Model
Source: Pathogens. 2026 Oct 9;15(10):1071. doi: 10.3390/pathogens15101071 (PMC13708033; doi:10.3390/pathogens15101071)
Supplement: Supplementary file 1 [file pathogens-15-01071-s001.zip › Figure S1 Alpha- and beta-diversity analyses of the intestinal bacterial community.pdf]

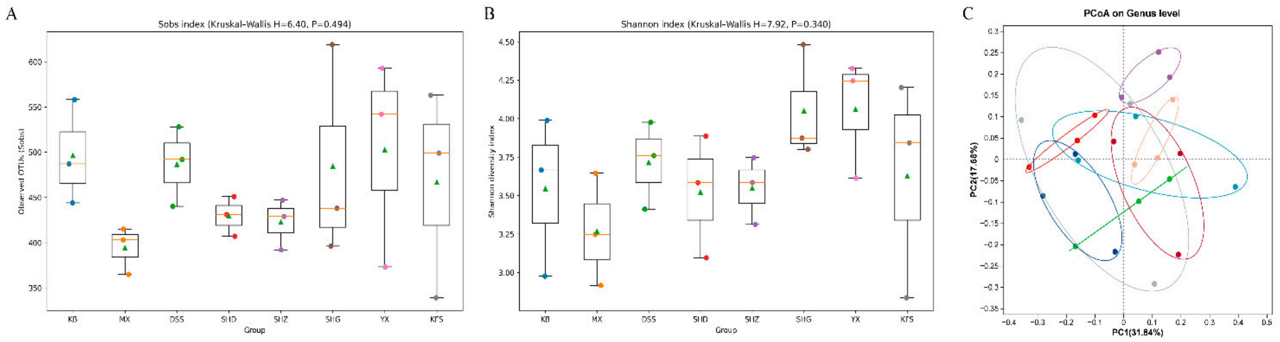

Figure S1. Alpha- and beta-diversity analyses of the intestinal bacterial community. (A) Observed OTU richness (Sobs); (B) Shannon diversity index; (C) principal coordinates analysis (PCoA) based on Bray–Curtis dissimilarity. No significant differences in Sobs or Shannon diversity were detected among groups. PERMANOVA indicated an overall difference in bacterial community composition among the eight groups ($F = 1.781$, $R^2 = 0.438$, $P = 0.011$). The group codes used in the microbiota plots correspond to the experimental groups as follows: KB, Control; MX, DSS + C.A (UCCA model); DSS, DSS-only group; KS, Caffeic Acid; SHD, SP-L; SHZ, SP-M; SHG, SP-H; and YX, Mesalazine.
