# Supplementary material for: Effects of Sanghuang Extract on Candida albicans Colonization and Intestinal Inflammation in a DSS-Induced Colitis Mouse Model
Source: Pathogens. 2026 Oct 9;15(10):1071. doi: 10.3390/pathogens15101071 (PMC13708033; doi:10.3390/pathogens15101071)
Supplement: Supplementary file 1 [file pathogens-15-01071-s001.zip › Figure S2. Representative SEM images of C. albicans after 24 h exposure to Sanghuang extract..pdf]

24h

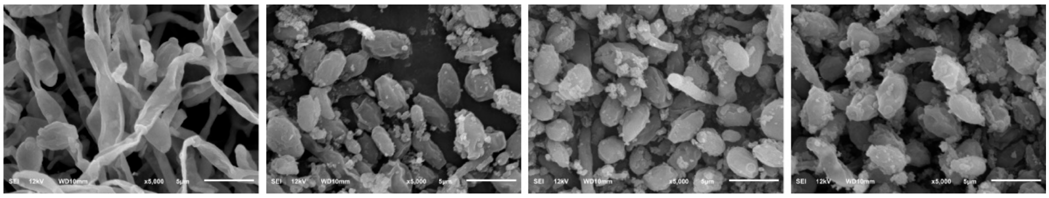

Figure S2. Representative scanning electron microscopy images of C. albicans after 24 h exposure to Sanghuang extract. (A) Untreated control; (B) 0.5×SMIC$_{80}$; (C) 1×SMIC$_{80}$; (D) 2×SMIC$_{80}$. Images are presented for qualitative morphological comparison.
