# Supplementary material for: Effects of Sanghuang Extract on Candida albicans Colonization and Intestinal Inflammation in a DSS-Induced Colitis Mouse Model
Source: Pathogens. 2026 Oct 9;15(10):1071. doi: 10.3390/pathogens15101071 (PMC13708033; doi:10.3390/pathogens15101071)
Supplement: Supplementary file 1 [file pathogens-15-01071-s001.zip › Figure S3 Representative ZO-1 immunohistochemical staining of colon tissues from the experimental groups..pdf]

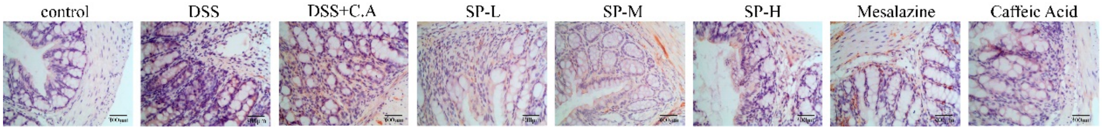

Figure S3. Representative ZO-1 immunohistochemical staining of colon tissues from the experimental groups. ZO-1 immunohistochemistry was performed and multiple microscopic fields were inspected to assess the staining pattern. The staining patterns were interpreted qualitatively. Original magnification: 200×.
